# Effects of Ta Addition on the Solidification and Homogenization of Ni-Based Superalloy GH4065A

**DOI:** 10.3390/ma19051002

**Published:** 2026-03-05

**Authors:** Wenyun Zhang, Linhan Li, Hongyu Su, Tong Wang, Ji Zhang, Yongquan Ning, Beijiang Zhang

**Affiliations:** 1 High Temperature Materials Research Division, Central Iron & Steel Research Institute, Beijing 100081, China; zhangwenyun@163.com (W.Z.); llhsuperalloy@163.com (L.L.); 011500003668@crrcgc.cc (H.S.); zhangji@cisri.cn (J.Z.); 2 Gaona Aero Material Co., Ltd., Beijing 100081, China; 3 The Key Laboratory of Electromagnetic Processing of Materials, Ministry of Education, Northeastern University, Shenyang 110819, China; wangtong@epm.neu.edu.cn; 4 School of Materials Science and Engineering, Northwestern Polytechnical University, Xi’an 710072, China; luckyning@nwpu.edu.cn

**Keywords:** Ni-based superalloy, Tantalum, solidification, segregation, homogenization, η phase

## Abstract

Increased alloying content in advanced Ni-based superalloys for large disc forgings intensifies microsegregation and promotes the formation of detrimental secondary phases, challenging the cast-and-wrought processing route. This study investigates the effects of Ta addition on the solidification and homogenization behaviors of a high-alloyed GH4065A superalloy by comparing the base alloy with a variant containing 5 wt.% Ta (5Ta alloy). As-cast and homogenized microstructures were characterized using SEM and EPMA, solidification behavior was analyzed via DSC, and homogenization kinetics were modeled. Results demonstrate that Ta addition stabilizes the η phase, increasing its solidification temperature and fraction in the as-cast microstructure, but does not alter the solidification sequence. During homogenization, Nb remained the most segregated element and governed the homogenization kinetics, whereas Ta preferentially partitioned into MC carbides and the η phase. The diffusion activation energy for Nb in the 5Ta alloy was determined, and a diffusion model was established to describe the elimination of microsegregation. Optimum homogenization parameters were determined to completely dissolve the η phase and eliminate microsegregation. The results indicate that strategic Ta addition for enhanced performance does not compromise ingot manufacturability, providing valuable guidance for the processing and composition design of advanced disc superalloys.

## 1. Introduction

Due to the advantages of high strength, good oxidation and corrosion resistance, as well as excellent microstructural stability at high temperatures, Ni-based superalloys are extensively used to manufacture discs for aerospace engines and various industrial gas turbine engines [1,2]. Recent rapid expansion in the gas turbine industry has increased the demand for larger, higher-performance superalloy disc forgings [3,4,5,6,7]. Therefore, many highly alloyed superalloys such as GH4065A, GH4720Li, and GH4175 alloys with a γ′ phase fraction larger than 40% have been utilized and processed via the cast-and-wrought (C&W) routine due to the advantages of lower production cost and higher efficiency when compared with the powder metallurgy method [8,9,10]. However, higher alloying content can cause severe segregation during the solidification process of large-scale ingots, leading to non-uniform microstructure and undesirable precipitation of the η phase, δ phase and TCP (Topologically Close-Packed) phases. These are detrimental to the hot workability of the ingot as well as the final mechanical properties of the forged product [11,12]. This situation presents a challenge to the C&W processing route, heightening the importance of homogenization treatment. The homogenization step remains critical because it is the primary process used to eliminate chemical segregation and dissolve undesirable precipitates of the ingot [13].

In the past few years, many researchers have extensively studied the microsegregation behavior of the as-cast microstructure to optimize homogenization treatment. Guo et al. [14] analyzed the microsegregation and homogenization behaviors of a GH3128 alloy produced by smelting. It was found that Al, W, and Cr were enriched in the interdendritic region, while Ni and Mo were concentrated in the dendritic core. After a two-step homogenization at 1130 °C for 12 h and 1210 °C for 36 h, the microsegregation in the ingot was effectively eliminated. In addition, it was reported that Co, Cr, and W were enriched in the dendritic arms of the GH4975 alloy, while Nb, Ti, Mo, and Al were enriched in the interdendritic region. The optimal homogenization process was determined to be 1220 °C for 10 h [15].

To enhance the mechanical performance, the alloying content of many advanced superalloys is intentionally increased by adding more γ′-forming elements (such as Al, Ti, Nb) as well as solution-strengthening elements (such as W, Co, Cr and Mo) [16,17,18]. For example, more Al, Ti, W, and Mo are added to GH4169D alloy for a higher operating temperature of 700 °C compared to the GH4169 alloy [19]. In addition, the incorporation of Ta element has emerged as a significant strengthening strategy in novel alloy design, as it is known to simultaneously enhance the matrix and promote the formation of the strengthening phase [20]. For example, third-generation directionally solidified single-crystal superalloys are normally designed to contain 4~12 wt.% of Ta to achieve excellent performance [21]. The addition of Ta can suppress the formation of TCP phases, refine the γ′ phase, and stabilize secondary carbides during long-term thermal exposure, thereby enhancing the mechanical properties of superalloys [22,23]. However, there is limited in-depth research on the solidification and homogenization behaviors of Ta-containing, highly alloyed disc superalloys. Therefore, the primary objective of this study is to investigate the influence of Ta on these behaviors.

GH4065A alloy is a highly alloyed C&W Ni-based superalloy that contains no Ta in its nominal composition, as shown in Table 1. It has a relatively high content of Al and Ti as γ′-forming elements, enabling the alloy to maintain high strength during prolonged service at temperatures up to 750 °C [8]. In this study, the effects of Ta addition on the solidification and homogenization behaviors of GH4065A alloy were investigated, based on the as-cast ingots of the alloy and a modified variant containing 5 wt.% Ta (5Ta alloy). Furthermore, the evolution of microstructure and elemental segregation conditions following various homogenization treatments was examined for both alloys, and the homogenization kinetics were determined based on a diffusion model. This work elucidates the influence of Ta addition on the solidification behavior of highly alloyed disc superalloys and offers guidance for optimizing the homogenization treatment, thereby informing the future improvement of alloy composition design.

## 2. Materials and Methods

The ingots of GH4065A alloy and 5Ta alloy with diameters of around 406 mm were produced by triple smelting process consisting of vacuum induction melting (VIM), electroslag remelting (ESR) and vacuum arc remelting (VAR). During VIM, electrodes with a diameter of 250 mm were cast under a vacuum level of <2.7 Pa and a leak rate of <2.7 Pa/min, with the refining and pouring temperatures set at 1550 °C and 1450–1480 °C, respectively. The subsequent ESR process produced 310 mm diameter ingots at a melting rate of 3.0–3.5 kg/min under Argon protection. Finally, VAR was performed to obtain 406 mm diameter ingots with a melting rate of 2.8–3.2 kg/min. The nominal compositions are shown in Table 1. It should be noted that the C content was also increased to 0.05% (wt.%) in the 5Ta alloy when compared with the base alloy.

To study the as-cast microstructure, cubic specimens (15 × 15 × 10 mm) were extracted from the mid-radius (1/2R) region of the ingot via wire electrical discharge machining. Homogenization treatments were subsequently applied to specimens taken from the 1/2R location. These specimens were heat-treated at temperatures of 1140, 1160, 1180, 1200, and 1220 °C for durations of 5, 10, 20, 40, and 80 h, respectively. To avoid cracking caused by rapid heating, the samples were initially preheated to 900 °C and then gradually heated to the target homogenization temperature at a controlled rate of 10 °C/h. Following homogenization, all specimens were air-cooled (AC).

The as-cast and homogenized microstructures were investigated by means of an Olympus GX71 optical microscope (OM, Tokyo, Japan) and a JSM-7800F field emission scanning electron microscope (SEM, Tokyo, Japan). The composition of the precipitates was determined using energy-dispersive spectroscopy (EDS). The specimens were ground using SiC papers with grit sizes of 120, 320, 800, 1500 and 2000, respectively. Mechanical polishing was performed with a 2.5 μm diamond spray polishing agent. To reveal the dendrites, the specimens were etched in a solution composed of 100 mL alcohol, 100 mL HCl and 20 g CuCl_2_. For the SEM analysis, the mechanically polished specimens were electro-polished and electro-corroded at a DC voltage of 20 V and 5 V, respectively. The solution for electro-polishing was 80 vol.% CH_3_OH and 20 vol.% alcohol. The solution for electro-corroding consisted of 15 g CrO_3_, 10 mL H_2_SO_4_, and 170 mL H_3_PO_4_. After mechanical polishing, the elemental distribution of regions of interest was examined using a JXA-8350F field emission electron probe microanalysis (EPMA) operated at an accelerating voltage of 15 kV.

Differential scanning calorimetry (DSC) was employed to determine the dissolution temperatures of the precipitate phases in the specimens extracted from the 1/2R position of the ingots. The DSC procedure involved heating the specimen to 1400 °C at a rate of 5 °C/min, maintaining this temperature for 3 min, and subsequently cooling to 1000 °C at the same rate. Throughout the entire process, high-purity argon gas (99.99%) was continuously supplied to the chamber to prevent oxidation. The equilibrium phase diagrams were calculated using the Ni-based superalloy database of the JMatPro software V7.0 (Guildford, UK).

## 3. Results

### 3.1. Thermodynamic Predictions

**Figure 1:**
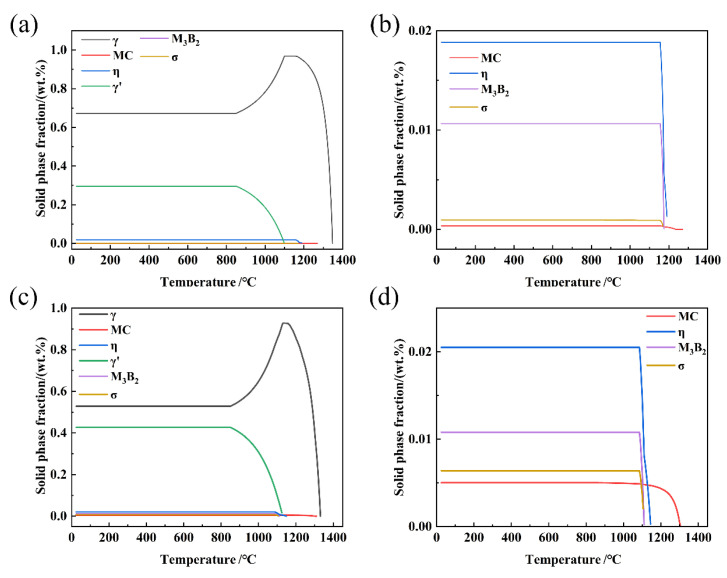
Phase diagrams for the (**a**) base alloy with (**b**) partial magnification of (**a**); (**c**) the 5Ta alloy with (**d**) partial magnification of (**c**).

Figure 1 presents the thermodynamic predictions for the base alloy and the 5Ta alloy calculated by JMatPro software based on the compositions listed in Table 1. A comparison of Figure 1a,c reveals that the addition of Ta increases both the phase fraction and solvus temperature of γ′ precipitates. Similarly, from the comparison between Figure 1b,d, it is predicted that Ta addition enhances the phase fractions of the η and σ phases but has no significant effect on the M_3_B_2_ boride phase.

### 3.2. As-Cast Microstructure

**Figure 2:**
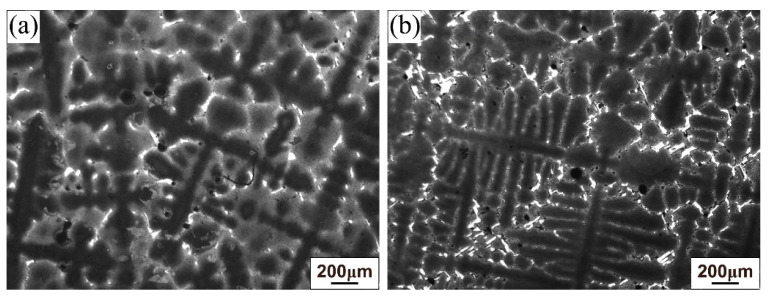
BSE images of as-cast microstructure of (**a**) base alloy ingot and (**b**) 5Ta alloy ingot at 1/2R position.

Figure 2a,b show the backscatter electron (BSE) images of the as-cast microstructure at mid-radius (1/2R) position of the base alloy ingot and 5Ta alloy ingot, respectively. The dark contrast regions correspond to a typical dendritic microstructure, while the brighter contrast areas represent the interdendritic zones containing precipitates. A significantly greater number of interdendritic precipitates exhibiting bright contrast was observed in the 5Ta alloy compared to the base alloy.

**Figure 3:**
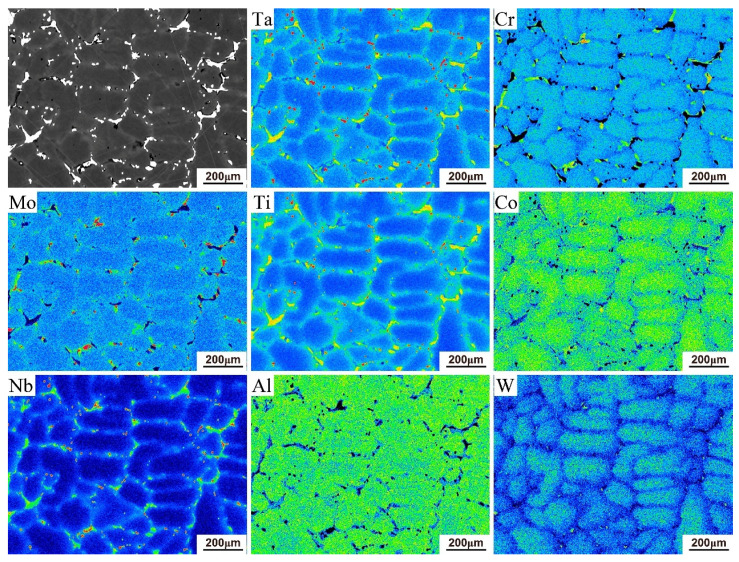
Element distribution maps of the as-cast microstructure of 5Ta alloy.

**Figure 4:**
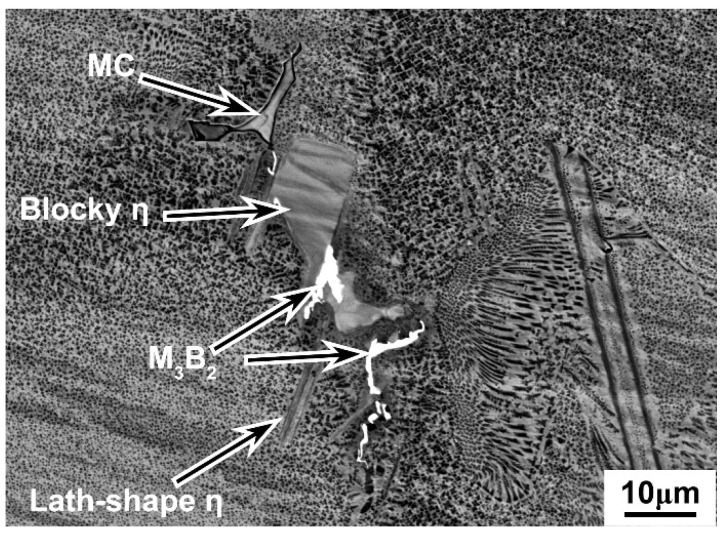
Precipitates in the as-cast microstructure of 5Ta alloy.

The elemental distributions of the as-cast microstructure of both alloys were further analyzed by EPMA. The elemental distribution maps in Figure 3 illustrate the partitioning of constituent elements, with concentration levels differentiated using a rainbow color scale. Low to high elemental concentrations are represented by a progression from dark blue to green, yellow, and finally red. As shown in Figure 3, Ta was observed to segregate in the interdendritic regions of the 5Ta alloy, in a manner similar to Nb and Mo. Furthermore, the as-cast microstructure of the 5Ta alloy was found to consist of three distinct phases (Figure 4), with Ta exhibiting different partitioning behaviors among them, as revealed by the elemental distribution maps (Figure 3) and EPMA point analysis (Table 2). Specifically, Ta preferentially partitioned into MC carbides, which also contained the highest concentrations of Ti and Nb. Additionally, Ta was detected in the blocky and lath-shaped η phases, which were enriched in Ti, Nb, and Al but depleted in Mo. In contrast, Ta was largely excluded from the M_3_B_2_ boride, which was characterized by relatively high Mo, Nb, and Cr contents.

**Figure 5:**
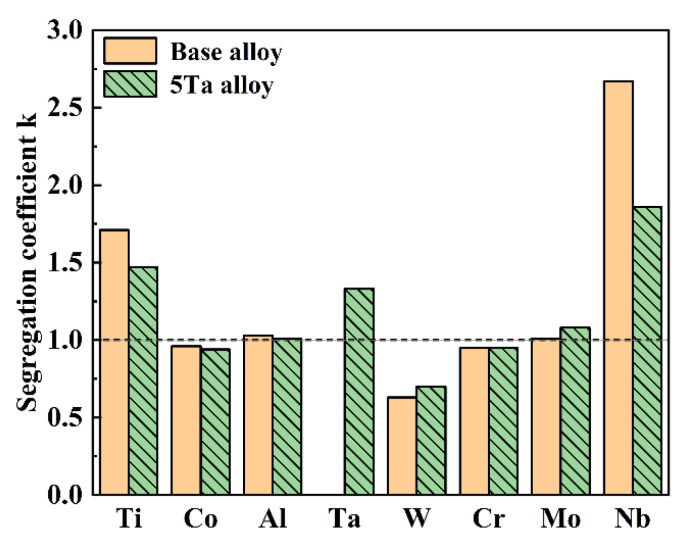
Segregation coefficient *K* of key alloying elements in both the base alloy ingot and the 5Ta alloy ingot.

To assess the extent of elemental segregation, EPMA point analysis was employed on the samples extracted from the 1/2R position of the base alloy ingot and the 5Ta alloy ingot. The weight percentages of key alloying elements (Ti, Co, Al, Ta, W, Cr, Mo, and Nb) were measured separately at the dendritic cores and interdendritic regions. Then their segregation coefficient K was calculated using the following ratio:

(1) $$K=w_{J}/w_{G}$$

where *w*_J_ and *w*_G_ are the average weight percentages of the element in the interdendritic and the dendritic regions, respectively. According to Equation (1), a *K* value approaching unity indicates a relatively uniform elemental distribution with negligible segregation. A value of *K* > 1 signifies that the element is enriched in the interdendritic regions, whereas *K* < 1 implies preferential segregation to the dendritic cores. As shown in Figure 5, the calculated segregation coefficients for the 5Ta alloy ingot indicate that Nb, Ti, Ta, and Mo exhibit *K* > 1, reflecting their enrichment in the interdendritic regions as positive segregation. In contrast, W, Cr, and Co display *K* < 1, corresponding to their preferential concentration within the dendritic cores as negative segregation. Among these elements, Nb, Ti, Ta, and W show the most pronounced segregation behavior. A comparison between the base alloy and the 5Ta alloy shows that, apart from Ta, only Nb exhibits a significant difference in its segregation coefficient.

**Figure 6:**
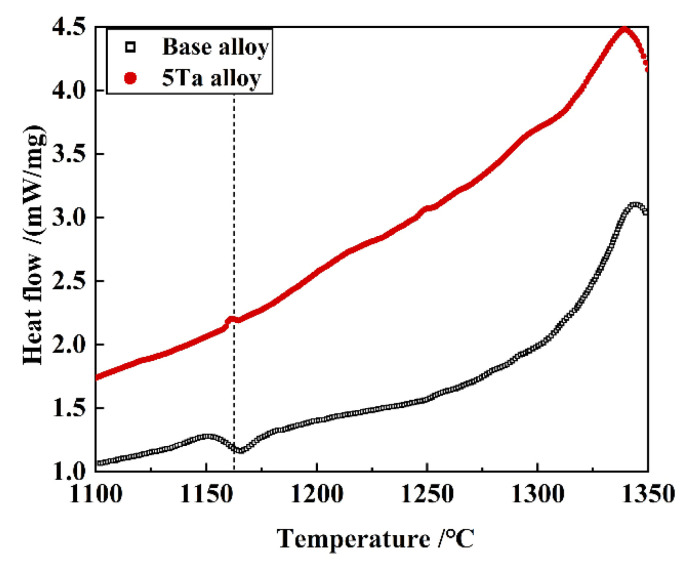
Cooling curves of the DSC specimens extracted from the base alloy ingot and the 5Ta alloy ingot, respectively.

Figure 6 shows the DSC cooling curves of the specimens extracted from the base alloy ingot and the 5Ta alloy ingot, respectively. Both alloys exhibit peaks in the temperature range of 1125 to 1165 °C, which correspond to phase precipitation during the cooling. It should be noted that the onset temperature of this precipitation process slightly differs between these two alloys, as the precipitation starts at a higher temperature in the 5Ta alloy.

### 3.3. Microstructure Evolution During the Homogenization

**Figure 7:**
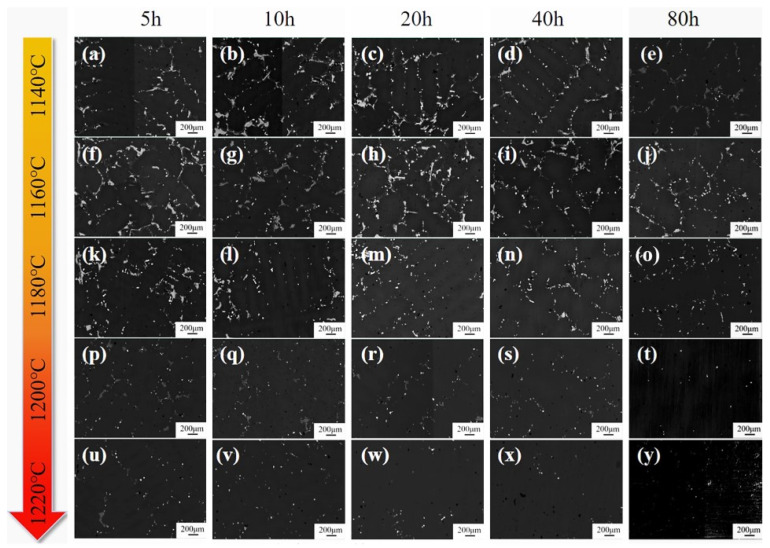
(**a**–**y**) BSE images of the 5Ta alloy following homogenization at various temperatures and time durations.

The evolution of the as-cast microstructure in the 5Ta alloy following homogenization at various temperatures and durations was examined using BSE imaging (Figure 7). In these images, the white contrast corresponds to dendritic regions, whereas the dark contrast represents the interdendritic areas containing precipitated phases. The dendritic structure progressively dissolved with increasing temperature or holding time. Complete dissolution was essentially achieved after 80 h at 1180 °C (Figure 7o). Following homogenization at 1200 °C and 1220 °C, the fraction of interdendritic precipitates was dramatically reduced, even after 20 h (Figure 7r) and 10 h (Figure 7v), respectively.

**Figure 8:**
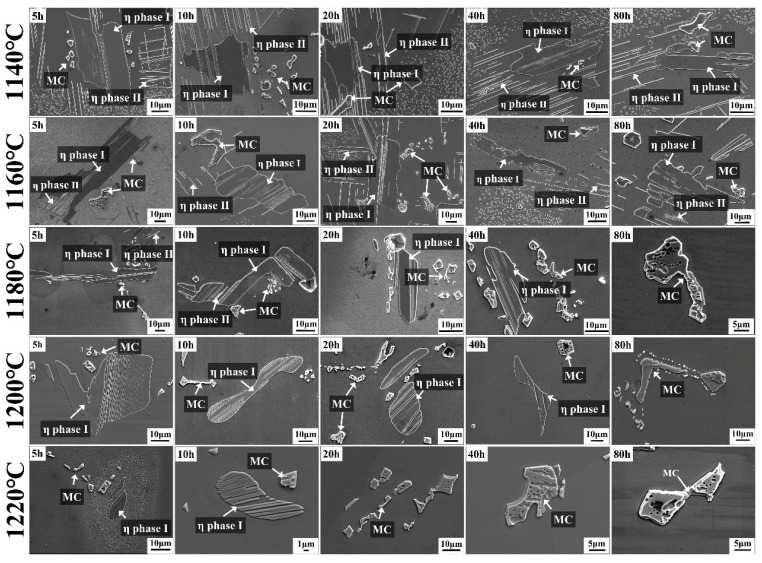
SEM images of the 5Ta alloy illustrating phase evolution under various homogenization treatments.

The phase evolution in the 5Ta alloy during homogenization at various temperatures and durations is detailed in Figure 8. As identified in the as-cast state (Figure 4), the initial microstructure comprised blocky η phase I, lath-shaped η phase II, as well as irregular MC carbides and M_3_B_2_ borides. Subsequent homogenization treatments at 1140 °C did not eliminate these phases, all of which persisted even after 80 h. At 1160 °C, the lath-shaped η phase II dissolved after 40 h, while the blocky η phase I remained after 80 h. Treatment at 1180 °C led to the dissolution of the lath-shaped η phase II by 20 h. Upon extending the holding time to 80 h at this temperature, both η phase variants dissolved, leaving only the MC and M_3_B_2_ phases. At the higher temperatures of 1200 °C and 1220 °C, the lath-shaped η phase II dissolved within 5 h. Complete dissolution of the blocky η phase I was achieved after 80 h at 1200 °C and 20 h at 1220 °C.

**Figure 9:**
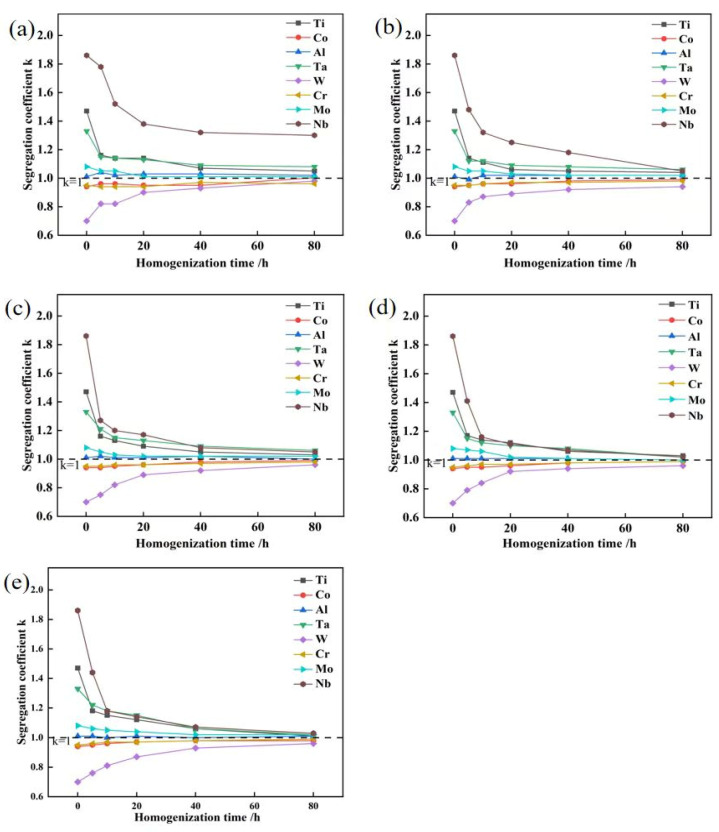
Segregation coefficients (K) of key alloying elements following homogenization at (**a**) 1140 °C, (**b**) 1160 °C, (**c**) 1180 °C, (**d**) 1200 °C, and (**e**) 1220 °C for varying durations.

**Figure 10:**
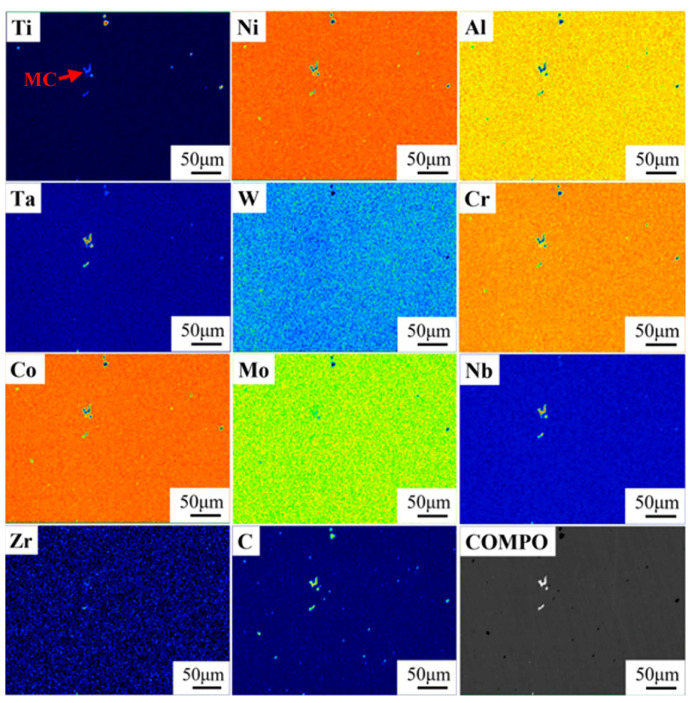
EPMA elemental distribution maps of 5Ta alloy homogenized at 1180 °C for 80 h.

The weight percentages of the key alloying elements Ti, Co, Al, Ta, W, Cr, Mo and Nb were measured in both the interdendritic and dendritic regions following various homogenization treatments. The corresponding segregation coefficient, *K*, calculated via Equation (1), is plotted in Figure 9, and the corresponding segregation coefficients for the baseline GH4065A can be found in our previous study [24]. The K values for all elements decreased most rapidly in the initial stage and progressively converged toward unity with extended time, indicating a gradual elimination of microsegregation. Notably, Nb exhibited the most pronounced segregation in the interdendritic region and maintained this highest level throughout the homogenization process. A similar trend was observed for W in the dendritic region. In particular, the K value of Nb remained as high as 1.3 even after 80 h at 1140 °C (Figure 9a). In addition, Figure 10 presents the elemental distribution of the 5Ta alloy following homogenization at 1180 °C for 80 h, as determined by EPMA analysis. It is observed that the alloying elements are distributed nearly uniformly, with only Ti-, Ta-, and Nb-enriched MC carbides present.

## 4. Discussion

### 4.1. Effects of Ta on the Solidification Behavior

Based on thermodynamic predictions (Figure 1) and as-cast microstructural observations (Figure 2), the solidification sequence of both the base alloy and 5Ta alloy is determined to follow the same pathway: Liquid → γ matrix → MC → η phase → M_3_B_2_ boride. The addition of Ta does not alter the types of precipitates formed in the as-cast microstructure. Instead, Ta primarily influences the fraction and solidification temperature of specific phases. In the 5Ta alloy, the increased carbon and Ta content lead to a higher fraction of MC carbides. As confirmed by EPMA point analysis (Table 2), Ta is strongly partitioned into the MC carbides, where its concentration exceeds that in all other phases, consistent with its well-known role in stabilizing the carbide [25]. Following the solidification of MC carbides, Co-rich η precipitates exhibiting blocky (η-I) and lath-shaped (η-II) morphologies are formed in the interdendritic regions, as depicted in Figure 4. DSC testing results (Figure 6) indicate that the measured onset precipitation temperature of η phase (~1165 °C) is higher in the 5Ta alloy than in the base alloy. Furthermore, a larger fraction of η phase is observed in the as-cast microstructure of the 5Ta alloy (Figure 2), which aligns with the thermodynamic predictions in Figure 1. Therefore, Ta promotes the formation of η phase during the solidification process. This stabilizing effect of Ta on the η phase is consistent with previous reports for other Ni-base superalloys [26]. Regarding the M_3_B_2_ boride, EPMA point analysis did not detect Ta (Table 2), indicating that the addition of Ta has a negligible influence on the formation of M_3_B_2_ boride.

### 4.2. Phase Evolution During the Homogenization

The determination of appropriate homogenization parameters for the 5Ta alloy requires a comprehensive consideration of segregation elimination, the dissolution of deleterious phases, and grain size control. Given that the η phase can serve as a nucleation site for cracks during deformation [27], its complete dissolution before cogging is essential. On the other hand, a small amount of sparsely distributed MC-type carbides is considered to have no adverse effect on the mechanical properties of the alloy [28]. Therefore, in the study of the homogenization behavior of the 5Ta alloy, the η phase was determined to be the primary phase requiring dissolution, while the MC carbides were not considered a priority.

During the homogenization process at 1140 °C (Figure 8), both the blocky η phase I and the lath-like η phase II persisted even after 80 h, whereas at 1160 °C, the lath-like η phase II dissolved after 40 h, while the blocky η phase I remained after 80 h. Treatment at 1180 °C led to the dissolution of both η phase variants with a holding time of 80 h. This suggests that the temperature required for complete η phase dissolution lies between 1160 °C and 1180 °C. This temperature is notably higher than the reported dissolution temperature of ~1140 °C for the η eutectic phase in a Ta-free Ni-Co-based superalloy [29]. As displayed in Figure 7 and Figure 8, at the higher homogenization temperatures of 1200 °C and 1220 °C, the primary effect of prolonged holding shifts to the dissolution of MC carbides.

### 4.3. Segregation Elimination During the Homogenization

According to the segregation coefficients calculated in Figure 5 and Figure 9, Nb exhibited the most pronounced segregation in the as-cast state and maintained the highest level throughout homogenization. Although both Nb and Ta are slow-diffusing refractory elements, Nb remained the most strongly segregated element even with the addition of 5 wt.% Ta. This is attributed to the partitioning behavior of Ta, which is primarily concentrated in MC carbides and the η phase rather than in the matrix (Table 2). Consequently, Nb remains the key element controlling segregation elimination during the homogenization of the 5Ta alloy. Therefore, a diffusion kinetics analysis of Nb element during the homogenization is required.

**Figure 11:**
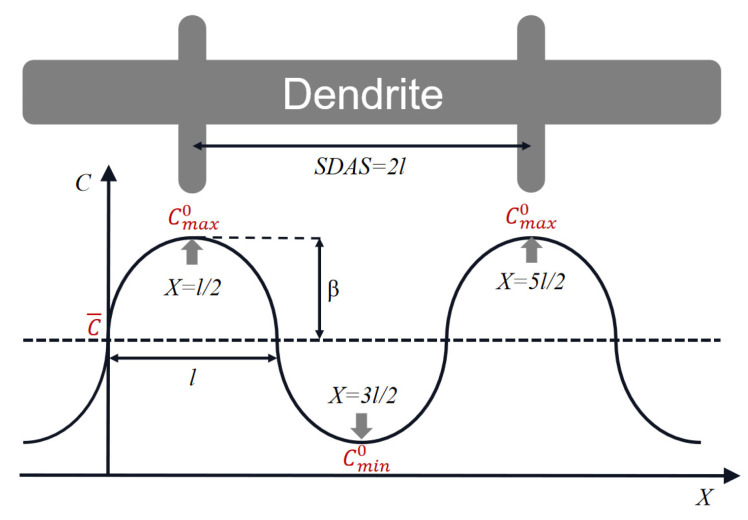
Schematic of the microsegregation of alloying elements between the dendrite arms [30].

Figure 11 shows the schematic of the microsegregation of alloying elements between the dendrite arms [30]. The elimination of microsegregation in the as-cast microstructure can be expressed by the following Equation (2):

(2) $$C=\bar{C}+\beta \sin\left(\pi x/l)exp(-\pi^{2}Dt/l^{2}\right)$$

where *C* is the concentration of the segregating element at time *t*, $\bar{C}$ is the average composition of the segregating element, β is the amplitude of the initial concentration profile, *D* is the interdiffusion coefficient of the element in the matrix, and *l* is half of the secondary dendrite arm space (SDAS).

Based on Figure 11, $C_{max}^{t}$ and $C_{min}^{t}$ can be determined at *X* = (4n − 3)*l*/2 and *X* = (4n − 1)*l*/2, respectively, where *n* is a natural number:

(3) $$C_{max}^{t}=\bar{C}+\beta exp(-\pi^{2}Dt/l^{2})$$

(4) $$C_{max}^{0}=\bar{C}+\beta$$

(5) $$C_{min}^{t}=\bar{C}-\beta exp(-\pi^{2}Dt/l^{2})$$

(6) $$C_{min}^{0}=\bar{C}-\beta$$

The residual segregation index (*δ*) is commonly employed to characterize the severity of elemental microsegregation during processes. In engineering applications, it is commonly accepted that microsegregation is effectively eliminated once the residual segregation index falls below 0.2 [31]. The residual segregation index (*δ*) for the homogenization treatment has been defined as follows [32]:

(7) $$\delta =\frac{C_{max}^{t}-C_{min}^{t}}{C_{max}^{0}-C_{min}^{0}}$$

where $C_{max}^{t}$ and $C_{min}^{t}$ are the highest and the lowest concentrations of the element, respectively, after homogenization for time *t*. $C_{max}^{0}$ and $C_{min}^{0}$ are the highest and the lowest concentrations of the element in the as-cast alloy. The residual segregation index of Nb (*δ*_Nb_) after homogenization at different temperatures and time durations was calculated based on Equation (7), and the results are shown in Table 3. It can be seen that at a given temperature, the *δ*_Nb_ decreased with the increasing homogenization time. In this study, the microsegregation of Nb in the 5Ta alloy is considered to be effectively eliminated following homogenization at 1180 °C for 80 h, which is confirmed by the EPMA mapping analysis shown in Figure 10.

**Figure 12:**
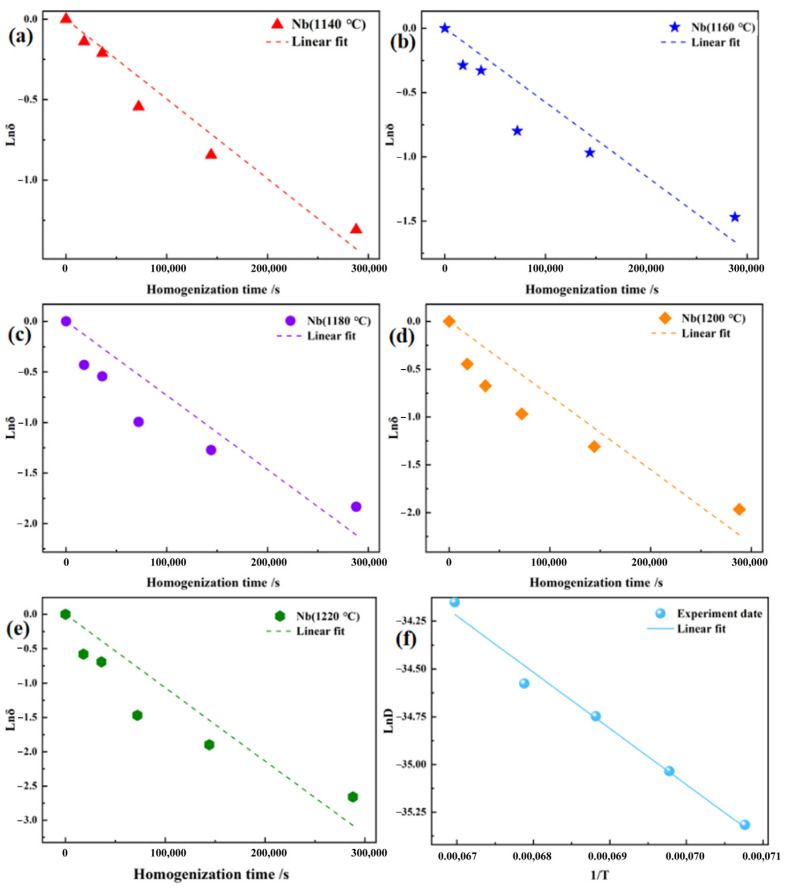
Relationship between ln*δ* and *t* at various temperatures: (**a**) 1140 °C, (**b**) 1160 °C, (**c**) 1180 °C, (**d**) 1200 °C and (**e**) 1220 °C; and (**f**) the corresponding relationship between ln*D* and 1/T.

Based on Equation (8), the relationship between ln*δ*_Nb_ and the time *t* was calculated for temperatures of 1140, 1160, 1180, 1200 and 1220 °C respectively. These relationships were plotted and subjected to linear regression analysis, as shown in Figure 12a–e. The diffusion coefficient D at each temperature was determined from the slopes of these fitted lines. The calculated values are 4.59 × 10^−16^, 6.08 × 10^−16^, 8.12 × 10^−16^, 9.64 × 10^−16^ and 1.47 × 10^−15^ m^2^/s at 1140, 1160, 1180, 1200, and 1220 °C, respectively. These results demonstrate a clear increase in the diffusion coefficient of Nb with rising homogenization temperature.

Based on Equations (3)–(7), *δ* can also be expressed as follows:

(8) $$ln\delta =(-\pi^{2}D/l^{2})t$$

In addition, the relationship between diffusion coefficient D and the reciprocal temperature 1/T can be expressed by the following Arrhenius Equation (9) [33]:

(9) $$D=D_{0}exp(-\frac{Q}{RT}),$$

where D0 is the diffusion constant, Q is the diffusion activation energy, and R is the gas constant of 8.314 J/(mol·K). Taking the natural logarithm of Equation (9) yields the following expression:

(10) $$lnD=-\frac{Q}{RT}+lnD_{0}$$

According to the linear fitting shown in Figure 12f, the values of Q and D0 for Nb in the 5Ta alloy were calculated to be 244.485 kJ/mol and 4.94 × 10^−7^ m^2^/s, respectively. It is reported that the diffusion activation energy of other Ni-Nb systems was 234.1 kJ/mol [30]. The activation energy for Nb diffusion in the 5Ta alloy is higher than that in other Ni-Nb systems, which might be attributed to the enhanced diffusion resistance resulting from the increased content of Ta or other alloying elements.

Based on the above results, the relationship between the residual segregation index *δ*, the secondary dendrite spacing SDAS, the homogenization temperature T, and the homogenization time *t* for Nb diffusion in the 5Ta alloy can be established by combining Equations (7) and (8) as follows:

(11) $$\delta =\exp\left[-\frac{\left(4.94\times 10^{-7}\right)\pi^{2}}{l^{2}}\exp(-\frac{244,485}{RT})t\right]$$

According to the above diffusion model, the optimal homogenization time can be predicted by setting the residual segregation index to 0.2 for a given secondary dendrite spacing and homogenization temperature.

In summary, based on the study of phase evolution and segregation, the optimal homogenization treatment for the GH4065A alloy with 5 wt.% Ta addition is determined to be 1180 °C for 80 h. This parameter ensures the complete dissolution of the η phase and the effective elimination of microsegregation. Higher temperatures (e.g., 1200 °C) risk incipient melting and grain coarsening, which would detrimentally affect hot workability. Furthermore, the results demonstrate that Ta addition does not significantly alter the solidification behavior. Nb remains the rate-controlling element for segregation elimination during homogenization. Therefore, the strategic addition of Ta to enhance mechanical properties is not expected to adversely impact ingot production or subsequent homogenization processes.

## 5. Conclusions

In this study, the effects of Ta addition on the solidification and homogenization behaviors of Ni-based superalloy GH4065A were investigated. The main conclusions are as follows:

(1) The addition of Ta does not change the solidification sequence of the alloy. Ta strongly partitions into MC carbides and η precipitates but is depleted in M_3_B_2_ borides. It primarily increases the volume fraction and the solidification temperature of the η phase, thereby stabilizing it. Homogenization at 1140 °C and 1160 °C fails to completely dissolve the η phase, whereas homogenization at 1180 °C or higher achieves full dissolution.
(2) Despite the addition of 5 wt.% Ta, Nb remains the most strongly segregated element in the alloy, exhibiting the highest segregation coefficient (*K*) in the as-cast state and maintaining this predominance throughout homogenization. This persistence can be attributed to the partitioning behavior of Ta, which primarily concentrates in MC carbides and the η phase rather than in the matrix.
(3) The diffusion activation energy *Q* and the diffusion constant *D*_0_ of Nb in the 5Ta alloy were 244.485 kJ/mol and 4.94 × 10^−7^ m^2^/s, respectively. The diffusion model describing the homogenization kinetics of Nb can be expressed as follows:
  $$\delta =exp\left[-\frac{\left(4.94\times 10^{-7}\right)\pi^{2}}{l^{2}}\exp\left(-\frac{244,485}{RT}\right)t\right]$$
(4) The optimal homogenization parameters for this 5Ta alloy are determined to be 1180 °C for 80 h, which achieves complete dissolution of the η phase and effectively eliminates microsegregation.

## Figures and Tables

**Table 1:** Nominal compositions of GH4065A base alloy and 5Ta alloy (wt.%).

Alloy	Co	Cr	Al	Ti	Nb	Ta	Mo	W	C	Zr	B	Ni
Base alloy	13.0	16.0	2.1	3.7	0.7	0	4.0	4.0	0.01	0.045	0.015	Bal.
5Ta alloy	13.0	16.0	2.1	3.7	0.7	5	4.0	4.0	0.05	0.045	0.015	Bal.

**Table 2:** EPMA point analysis results of different phases in the as-cast microstructure of 5Ta alloy (wt.%).

Phase	Co	Cr	Al	Ti	Nb	Ta	Mo	W	C	Zr	B	Ni
MC	0	0.9	0.4	6.8	31.3	18.5	1.2	3.3	18.0	4.9	0	3.5
Blocky η phase	12.5	2.5	2.4	5.7	4.2	10	0.7	2.9	1.4	0	0	54.2
Lath-shaped η phase	13.6	5.3	2.7	5.0	5.2	13	1.1	3.7	2.3	0	0	50.8
M_3_B_2_ boride	6.4	22.7	0.3	3.1	5.1	0	29.0	10.9	0.5	0	4.0	17.6

**Table 3:** Residual segregation index of Nb under various homogenization treatments.

	Temperature/°C	1140	1160	1180	1200	1220
Time/h						
0		1	1	1	1	1
5		0.87	0.75	0.65	0.64	0.56
10		0.81	0.72	0.58	0.51	0.50
20		0.58	0.45	0.37	0.38	0.23
40		0.43	0.38	0.28	0.27	0.15
80		0.27	0.23	0.16	0.14	0.07

## Data Availability

The original contributions presented in this study are included in the article. Further inquiries can be directed to the corresponding author.
